# Social Network as Outbreak Investigation Tool

**DOI:** 10.3201/eid1709.110088

**Published:** 2011-09

**Authors:** Julia F. Howland, Craig Conover

**Affiliations:** Author affiliation: Illinois Department of Public Health, Chicago, Illinois, USA

**Keywords:** disease outbreaks, data collection, social network, investigation tool, Facebook, Illinois, gastrointestinal illness, email, letter

**To the Editor:** The recent article by Oh et al. (*1*) discussed the utility of email surveys for the investigation of outbreaks. After they have been created, digital surveys require less time to administer than paper-based or telephone surveys and can produce high-quality and timely data. During an outbreak in Illinois, we used email and a social networking site to distribute a link to a confidential Inquisit (www.millisecond.com) survey and compared characteristics of the groups that responded to each.

In December 2010, the Illinois Department of Public Health received a report of an outbreak of gastrointestinal illness among guests at a wedding reception. Health department staff converted a standard foodborne outbreak questionnaire to a digital format. The survey link was then distributed to guests by 2 methods: email from the reception hosts and the note function on the host’s Facebook page. Facebook has 500 million active users, 50% of whom check their Facebook pages every day (*2*). The Facebook note function is a blogging feature through which users can publish content visible to linked friends.

A total of 14 persons responded to the email-distributed survey link and 41 to the Facebook-distributed survey link. For each survey, data quality was high and response rates for questions were >90%. Facebook respondents were younger than email respondents (mean ages 29.8 and 37.4 years, respectively). Information provided by Facebook respondents covered persons 11 months to 80 years of age and by email respondents 1–67 years of age. Parents were asked to complete surveys for any children unable to answer the questions independently. The Facebook-distributed survey had a higher percentage of male respondents (41.5%) than did the email-distributed survey (21.4%).

Facebook-distributed surveys were answered significantly faster than email-distributed surveys (p<0.05). The mean number of hours from distribution to response was 42.3 for the email survey and 8.7 for the Facebook survey. The Facebook survey link was distributed at 6:00 pm on a Thursday evening; 34 (82.9%) surveys were completed by 9:00 am on Friday morning. On the basis of these responses, health department staff were able to identify the implicated foods the day after the questionnaires were distributed.

Distributing foodborne outbreak questionnaires through Facebook generated data that were complete and timely. Facebook-distributed surveys captured a wide range of respondent age groups and more male respondents than did email-distributed surveys. Previous studies of online survey response rates found rates to be significantly higher for women than for men (*3*). In addition to low cost and significantly improved survey response times, social networking distribution holds other advantages for health departments. Recall errors are reduced by distributing the survey to persons simultaneously and immediately. Posting of surveys through a health department’s social networking accounts could also enable participation of persons for whom the health department does not have contact information. Given these advantages and the widespread use of social networking, use of these tools should be considered as an option for survey distribution during outbreak investigations.
